# Dataset of reference values for the quantification of net primary production use (or biotic resource use) of feed ingredients in life cycle assessment

**DOI:** 10.1016/j.dib.2025.111929

**Published:** 2025-07-28

**Authors:** Samuel Le Féon, Joël Aubin, Aurélie Wilfart

**Affiliations:** INRAE, Institut Agro, SAS, 65 rue de Saint Brieuc, 35042 Rennes, France

**Keywords:** Environmental impact assessment, Animal nutrition, Aquaculture, Trophic level, NPPU, BRU

## Abstract

In order to quantify the pressure of livestock feeding practices on biotic resources with Life Cycle Assessment (LCA), Papatryphon et al. (2004) developed a midpoint impact category called Net Primary Production Use (NPPU). It is expressed in kg of carbon by kg of product and is derived from the carbon-content of the feed ingredient, adjusted by its trophic level following the formula developed by Pauly and Christensen (1995). Another name is found in literature for NPPU as Biotic Resource Use (BRU), Cashion et al. (2016) arguing that both are used for aquaculture, when BRU is also used for fisheries. Since, it has been widely used in LCA, especially to compare different feed solutions for aquaculture. NPPU has been calculated essentially for fish-based and plant-based feed ingredients. Despite the indicator was used in numerous publications for years, there is currently no proper database of reference values easy-to-use and validated by peers that includes a wide range of fish-based and plant-based feed ingredients. This data paper aims to produce this reference dataset of values to be used as standard in future studies. In addition, it also proposes values for other-animal-based feed ingredients, freshwater-fish-based feed ingredients and milk-based ingredients. Therefore, it is recommended to be used in future LCA studies of aquaculture. This data paper also provides the calculation rules and hypotheses made in the calculation, in order to explain the procedure, allowing readers to reproduce results and calculate values for their own ingredients. Finally, different levels (Tiers) of specificity are proposed for the datasets, that use more detailed primary data, associated with more uncertainty in return.

Specifications Table

**Table:** 

Subject	Earth & Environmental Sciences
Specific subject area	Environmental impact assessment of feed ingredients, in particular for animal nutrition
Type of data	The dataset comprises 11 *.CSV files for plant-based, marine-fish-based, freshwater-fish-based, other-animals-based and milk-based feed ingredients. All files contain raw and calculated data (i.e., NPPU derived from raw data). Raw data are provided for replication but not generated by the authors (collected on third party database, sources provided).
Data collection	The raw data used to calculate the NPPU were collected in various databases: - Tables of Composition and Nutritional Value of Feed Materials: Pig, Poultry, Sheep, Goats, Rabbits, Horses, Fish. INRA, Paris, also known as “Les Tables Vertes de l’INRA” [1] - Feed Tables for plant-based ingredients [2] - FishBase [3]. Missing data have been collected in literature. All sources are provided in the different CSV files.
Data source location	Storage of the data: - Institution: INRAE, SAS Join research unit - City/Town/Region: Rennes - Country: France - Latitude: 48° 6′ 48.51″N and longitude: 1° 40′ 32.55″W
Data accessibility	Repository name: https://entrepot.recherche.data.gouv.fr/ Data identification number: https://doi.org/10.57745/9YLZL9 Direct URL to data: https://entrepot.recherche.data.gouv.fr/dataset.xhtml?persistentId=doi:10.57745/9YLZL9
Related research article	No related research article

## 1 Value of the Data

• These data are useful to quantify the pressure of livestock feeding practices on biotic resources with life cycle assessment.
• This is the first and only homogeneous dataset of NPPU factors for both plant-based and marine fish-based ingredients.
• It includes in addition NPPU values for freshwater species, other animal-based ingredients and milk-based ingredients.
• It is useful to clarify past and future NPPU calculations, with a view of standardization as being pointed out in recent studies of aquaculture systems.
• Researchers, as well as any LCA practitioner may benefit from this dataset to enlarge the scope of LCA related to animal nutrition.
• Mostly used in LCA of aquaculture systems, NPPU could be applied to other animal productions (especially non-conventional species as insects), this being facilitated by this dataset.

## 2 Background

In order to quantify the pressure of livestock feeding practices on biotic resources with Life Cycle Assessment, Papatryphon et al. (2004) developed a midpoint impact category called Net Primary Production Use (NPPU). Since, it has been widely used in LCA, especially to compare different feed solutions for aquaculture. Therefore, it is recommended to be used in future LCA studies of aquaculture, but a need for standardization is pointed out. In most studies, by construction, feed is the only contributor to NPPU [[4], [5]]. NPPU has been calculated essentially for fish-based and plant-based feed ingredients. It is expressed in kg of carbon by kg of product and is derived from the carbon-content of the feed ingredient, adjusted by its trophic level following the formula developed by Pauly and Christensen (1995). NPPU is expressed in kg of carbon by kg of product. Another name is found in literature for NPPU as Biotic Resource Use (BRU), Cashion et al. [6] arguing that both are used, only for NPPU when BRU is also used for fisheries. Despite the indicator was used in numerous publications for years, there is currently no proper database of reference values easy-to-use and validated by peers that includes a wide range of fish-based and plant-based feed ingredients. For instance, in their study about emerging aquaculture systems [7], used NPPU values derived from [8] in order to allow replication and adhere to the need for standardisation of NPPU in LCAs pointed out by [6]. This need for standardization is also cited recently in literature [9]. In the same time [10], recommend the use of NPPU in future LCA studies of aquaculture systems. Recently, BRU values were proposed for fishmeal and fishoil for 16 marine species, including mass and economic allocations [11]. In this context, the present datapaper aims to clarify past and future calculations by making explicit raw data and derived NPPU factors. In addition, this dataset states the NPPU values used by the Ecoalim database, a national Dataset of Environmental Impacts of Feed Ingredients Used in French Animal Production [12]. Furthermore, recent study underlined the interest of animal coproducts to reduce NPPU impact in environmental friendly fish diets [13]. In addition, it also proposes values for other-animal-based feed ingredients (as poultry feathers), freshwater-fish-based feed ingredients and milk-based ingredients. This data paper also provides the calculation rules and hypotheses made in the calculation, in order to explain the procedure, allowing readers to reproduce the results and calculate values for their own ingredients. Different levels of specificity (Tiers) are proposed for the datasets, that use more detailed primary data, associated with more uncertainty in return (the mentioned uncertainty is not formally calculated in the present dataset).

## 3 Data Description

The dataset comprises 11 different *.CSV files for plant-based, marine-fish-based, freshwater-fish-based, other-animals-based and milk-based feed ingredients. Every file contains raw and calculated data (i.e., NPPU derived from raw data). Raw data are provided for replication but not generated by the authors (collected on third database, sources provided).

In more details, the dataset comprises:

- 1_Plant-based_NPPU.csv: this file is a one-sheet table with 11 columns:
  ○ The name of the feed, translated in English language as normalized by Feedbase database;
  ○ The name of the feed ingredient, as named in French in the original databases;
  ○ The category of the feed ingredient (as classified in the Feed Tables database (INRAE et al., 2017));
  ○ The crude protein content in the ingredient (extracted from two raw databases ([1]; INRAE et al., 2017));
  ○ The lipids content in the ingredient (extracted from two raw databases ([1]; INRAE et al., 2017));
  ○ The ashes content in the ingredient (extracted from two raw databases ([1]; INRAE et al., 2017));
  ○ The percentage of polysaccharides and hemicelluloses derived from previous data (see material and methods);
  ○ The carbon content (in g C / kg ingredient) calculated from previous data (see material and methods);
  ○ The NPPU (in g C / kg ingredient);
  ○ The NPPU (in kg C / kg ingredient);
  ○ The source used for raw data.
- 2.1_Marine_Fish_based_NPPU_fresh_Tiers1.csv, 2.2_Marine_Fish_based_NPPU_fresh_Tiers2.csv, 2.3_Marine_Fish_based_NPPU_fresh_Tiers3.csv: these three files are one-sheet tables with 19 columns:
  ○ Specie: the scientific name of the fish specie
  ○ Common_name: the name of the feed ingredient, in English language;
  ○ Common_name_FR: the name of the feed ingredient, in French language;
  ○ Ecosystem: the ecosystem (i.e., geographical area) used for Transfer Efficiency coefficients;
  ○ Trophic_level: the trophic level of the considered specie or group of species;
  ○ Trophic_level_SD: the standard deviation of trophic levels;
  ○ Transfer_efficiency: the transfer efficiency coefficients;
  ○ Comment: potential commentaries about the data;
  ○ Moisture_ %: the percentage of water;
  ○ Protein_ %: the percentage of crude protein content;
  ○ Fat_ %: the percentage of lipids;
  ○ Ash_ %: the percentage of ash;
  ○ Carbohydrates_ %: the percentage of Carbohydrates;
  ○ Source_TL: bibliographic source used for trophic levels
  ○ Source_Body_Composition: bibliographic source used for body compositions
  ○ Source_TE: bibliographic source used for transfer efficiency
  ○ C_content: the carbon content (in g C/kg of dry matter)
  ○ NPPU_g/kg: the calculated Net Primary Production Use (in g C/kg of feed ingredient)
  ○ NPPU_kg/kg: the calculated Net Primary Production Use (in kg C/kg of feed ingredient)
- 3_Marine_Fish_based_NPPU_ingredients.csv: one-sheet table with 26 columns:
  ○ Specie: the scientific name of the specie
  ○ Common_name: the name of the feed ingredient, in English language;
  ○ Common_name_FR: the name of the feed ingredient, in French language;
  ○ NPPU_kg/kg: the calculated Net Primary Production Use (in kg C/kg of feed ingredient)
  ○ Generic and specific fish meal and oil yields (4 columns);
  ○ Fish meal and oil densities (2 columns)
  ○ NPPU with mass allocation (2 columns)
  ○ NPPU with energy allocation for the different Tiers and yields (12 columns)
- 4.1_Freshwater_Fish_based_NPPU_fresh_Tiers1.csv,4.2_Freshwater_Fish_based_NPPU_fresh_Tiers2.csv: these two files are one-sheet tables with 19 columns:
  ○ Specie: the scientific name of the specie
  ○ Common_name: the name of the feed ingredient, in English language;
  ○ Common_name_FR: the name of the feed ingredient, in French language;
  ○ Ecosystem: the ecosystem (i.e., geographical area) used for Transfer Efficiency coefficients;
  ○ Trophic_level: the trophic level of the considered specie or group of species;
  ○ Trophic_level_SD: the standard deviation of trophic levels;
  ○ Transfer_efficiency: the transfer efficiency coefficients;
  ○ Comment: potential commentaries about the data;
  ○ Dry_matter_ %: the percentage of dry_matter;
  ○ Moisture_ %: the percentage of water;
  ○ Protein_ %: the percentage of crude protein content;
  ○ Fat_ %: the percentage of lipids;
  ○ Ash_ %: the percentage of ash;
  ○ Carbohydrates_ %: the percentage of Carbohydrates;
  ○ Source_TL: bibliographic source used for trophic levels
  ○ Source_Body_Composition: bibliographic source used for body compositions
  ○ Source_TE: bibliographic source used for transfer efficiency
  ○ C_content: the carbon content (in g C/kg of dry matter)
  ○ NPPU_g/kg: the calculated Net Primary Production Use (in g C/kg of feed ingredient)
  ○ NPPU_kg/kg: the calculated Net Primary Production Use (in kg C/kg of feed ingredient)
- 5_Freshwater_Fish_based_NPPU_ingredients.csv: one-sheet table with 18 columns:
  ○ Specie: the scientific name of the specie
  ○ Common_name: the name of the feed ingredient, in English language;
  ○ Common_name_FR: the name of the feed ingredient, in French language;
  ○ NPPU_T1(kg/kg): the calculated Net Primary Production Use with Tiers 1 (in kg C/kg of feed ingredient)
  ○ NPPU_T2(kg/kg): the calculated Net Primary Production Use with Tiers 2 (in kg C/kg of feed ingredient)
  ○ Generic and specific fish meal and oil yields (4 columns);
  ○ Fish meal and oil densities (2 columns)
  ○ Energetic allocation factors for meal and oil (2 columns);
  ○ NPPU with mass allocation (4 columns)
  ○ NPPU with energy allocation for the different tiers and yields (4 columns)
- 6_Other_animal_based_NPPU_fresh.csv: one-sheet tables with 19 columns:
  ○ Specie: the scientific name of the specie
  ○ Common_name: the name of the feed ingredient, in English language;
  ○ Common_name_FR: the name of the feed ingredient, in French language;
  ○ Ecosystem: the ecosystem (i.e., geographical area) used for Transfer Efficiency coefficients;
  ○ Trophic_level: the trophic level of the considered specie or group of species;
  ○ Trophic_level_SD: the standard deviation of trophic levels;
  ○ Transfer_efficiency: the transfer efficiency coefficients;
  ○ Comment: potential commentaries about the data;
  ○ Dry_matter_ %: the percentage of dry_matter;
  ○ Moisture_ %: the percentage of water;
  ○ Protein_ %: the percentage of crude protein content;
  ○ Fat_ %: the percentage of lipids;
  ○ Ash_ %: the percentage of ash;
  ○ Carbohydrates_ %: the percentage of Carbohydrates;
  ○ Source_TL: bibliographic source used for trophic levels
  ○ Source_Body_Composition: bibliographic source used for body compositions
  ○ Source_TE: bibliographic source used for transfer efficiency
  ○ C_content: the carbon content (in g C/kg of dry matter)
  ○ NPPU_g/kg: the calculated Net Primary Production Use (in g C/kg of feed ingredient)
  ○ NPPU_kg/kg: the calculated Net Primary Production Use (in kg C/kg of feed ingredient)
- 7_Other_animal_based_NPPU_ingredients.csv: one-sheet table with 4 columns:
  ○ Ingredient: the name of the feed ingredient, in English language;
  ○ Specie: the animal specie
  ○ NPPU_T1(kg/kg): the calculated Net Primary Production Use with Tiers 1 (in kg C/kg of feed ingredient)
  ○ Comment: potential commentaries about the data;
- 8_Milk_based_NPPU.csv: one file with one-sheet table with 17 columns:
  ○ Ingredient: the name of the feed ingredient, in English language;
  ○ Ingredient_FR: the name of the feed ingredient, in French language;
  ○ Trophic_level: the trophic level of the considered ingredient;
  ○ Trophic_level_SD: the standard deviation of trophic levels;
  ○ Transfer_efficiency: the transfer efficiency coefficients;
  ○ Comment: potential commentaries about the data;
  ○ Moisture_ %: the percentage of water;
  ○ Protein_ %: the percentage of crude protein content;
  ○ Fat_ %: the percentage of lipids;
  ○ Ash_ %: the percentage of ash;
  ○ Carbohydrates_ %: the percentage of Carbohydrates;
  ○ Source_TL: bibliographic source used for trophic levels
  ○ Source_Body_Composition: bibliographic source used for body compositions
  ○ Source_TE: bibliographic source used for transfer efficiency
  ○ C_content: the carbon content (in g C/kg of dry matter)
  ○ NPPU_g/kg: the calculated Net Primary Production Use (in g C/kg of feed ingredient)
  ○ NPPU_kg/kg: the calculated Net Primary Production Use (in kg C/kg of feed ingredient)
- NPPU_SD: standard deviations of NPPU values at specie-level

## 4 Materials and Methods

**Fig. 1:**
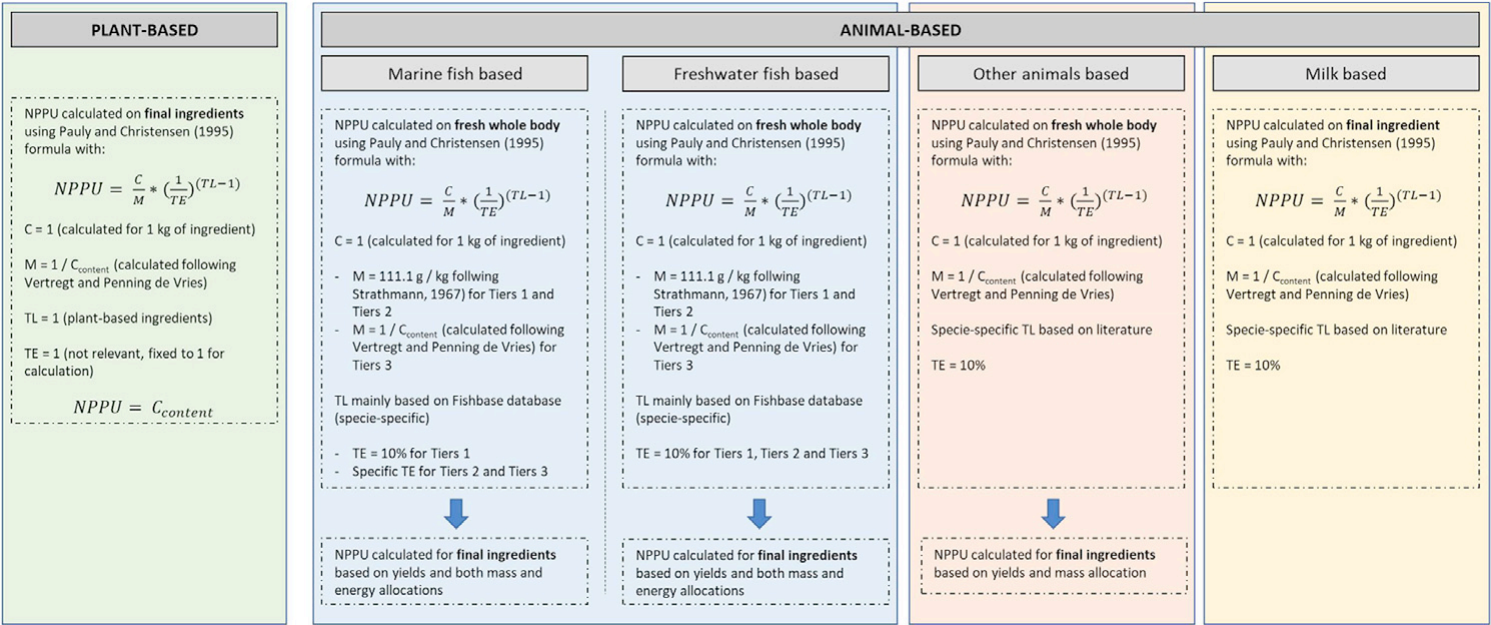
Adaptations to Pauly and Christensen formula, depending on the feed ingredient type.

To calculate NPPU values for feed ingredients, the initial formula from Pauly and Christensen (1995) and adapted in Cashion et al. [6] (Formula 1) is used. It is adapted depending on the type of feed ingredient (Fig. 1).

(1) $$NPPU=\;\frac{C}{M}*{\left(\frac{1}{TE}\right)}^{\left(TL-1\right)}$$

where, C is the quantity of ingredient, M is the ratio of wet weight biomass to carbon content, TE is the trophic transfer efficiency of the ingredient and TL is the trophic level of the ingredient.

This section details the methodological choices made for each type of ingredient (e.g., use of generic, specific or both TE).

For plant-based and milk-based ingredients, NPPU is directly calculated on final ingredient (e.g., rapeseed meal). For animal-based ingredients, NPPU is calculated on fresh ingredient (e.g., whole fresh anchovy) as some can be used as such. This allows users to apply their own allocation rules between co-products for a given specie (see recommendation section).

For users who prefer values at the ingredient level (e.g., fishmeal and fish oil), NPPU values were calculated using mass and energy allocation methods. Due to price volatility in years and between species, economic allocation was not used; instead, users are encouraged to apply their own economic values as appropriate. Some values can be found in Newton et al. [11].

1. Plant-based feed ingredients

Applying a trophic level of 1 for plant-based feed ingredients in Pauly and Christensen (1995), NPPU value for a given feed ingredient is equal to its carbon content in kg of carbon per kg of product. This is in accordance to Papatryphon et al. (2004). Then NPPU is equal to the sum of the carbon content of the fractions of proteins, lipids and carbohydrates (respectively proteins_content, lipids_content and carbohydrates_content in g/kg), following the formula 1, adapted from [14].

(2a) $$NPPU=\left[\begin{array}{c} C_{content}\left(carbohydrates\right)*carbohydrates\_content\\ +\;C_{content}\left(proteins\right)*proteins\_content\\ +\;C_{content}\left(lipids\right)*lipids\_content \end{array}\right]/1000$$

With:

$$C_{content}\left(proteins=C_{5}H_{7}O_{2}N\right)=\;\frac{5\;*\;M_{C}}{5\;*\;M_{C}\;+\;7\;*\;M_{H}\;+\;2\;*\;M_{O}\;+\;M_{N}}=0.531$$

$$C_{content}\left(lipids=C_{57}H_{104}O_{6}\right)=\;\frac{57*M_{C}}{57*M_{C}\;+104*M_{H}\;+6*M_{O}\;}=0.774$$

$$C_{content}\left(carbohydrates=C_{5}H_{8}O_{4}\right)=\;\frac{5*M_{C}\;}{5*M_{C}\;+8*M_{H}\;+4*M_{O}\;}=0.444$$

With:

**Table:** 

	Molar mass in g.mol^−1^

M_C_	12
M_H_	1
M_O_	16
M_N_	14

Then:

(2b) $$NPPU=\left[\begin{array}{c} 0.444*carbohydrates\_content\\ +\;0.531*proteins\_content\\ +\;0.774*\%lipids\_content \end{array}\right]/1000$$

The division by 1000 is made to obtain NPPU in kg C/kg.

2. Marine fish-based ingredients
  a. Fresh fishes
  For marine fish-based ingredients, three levels (Tiers) of NPPU were calculated for each specie from the most generic to the most specific
  — Generic NPPU (Tiers 1):
    ○ Generic TE: TE = 10 % [15].
    ○ Generic carbon content: The carbon content of 1 kg of fish is 111.1 g [16].
    ○ Specific TL [3,6].
  — Semi-specific NPPU (Tiers 2):
    ○ Specific TE: The 10 % average value can differ from an ecosystem to another, as well as temporally, following the interactions between ecosystems. Its value would range from 3.51 % to 38.1 % (average = 11.9 %) [17,18]. In Tiers 2 specific TE from literature were used [6,19].
    ○ Generic carbon content: The carbon content of 1 kg of fish is 111.1 g [16]. This can differ from a fish to another.
    ○ Specific TL [3,6].
  — Specific NPPU (Tiers 3):
    ○ Specific TE: The 10 % average value can differ from an ecosystem to another, as well as temporally, following the interactions between ecosystems. Its value would range from 3.51 % to 38.1 % (average = 11.9 %) [17,18]. In Tiers 2 specific TE from literature were used [6,19].
    ○ Specific carbon content: This can differ from a fish to another and is not equal to the generic 111.1 g/kg value. Specific values from literature were used [3,[20], [21], [22], [23], [24], [25], [26], [27]]
    ○ Specific TL [3,6].
b. Fishmeal and fish oil

Although the authors recommend to the users to use values for fresh species and apply own allocation rules (see recommendation section), NPPU have been calculated for one kilogram of fishmeal and fish oil using mass and energetic allocations, following Cashion et al. [6] (Formulas 3 and 4). As preconized in Cashion et al. [6], specific-yields were used. Nevertheless, NPPU were also calculated with generic yields to allow future comparisons. Several literature references were used for specific yields [6,11,28,29] and for energy densities of fishmeal and fish oil [6,30,31]. Generic values were used as a last resort when no specific values were found in literature. Considering the three tiers for fresh ingredients and generic/specific yields, six values of NPPU were calculated, respectively for generic yields (NPPU_T11, NPPU_T21 and NPPU_T31) and specific yields (NPPU_T12, NPPU_T22 and NPPU_T32).

(3) $$NPPU_{fishmeal}=\left(\frac{NPPU_{fresh}}{Yield_{meal}}\right)*\left(\frac{Yield_{meal}\;*\;energy_{meal}}{Yield_{meal}\;*\;energy_{meal}\;+\;Yield_{oil}\;*\;energy_{oil}}\right)$$

(4) $$NPPU_{fish\;oil}=\left(\frac{NPPU_{fresh}}{Yield_{oil}}\right)*\left(\frac{Yield_{oil}\;*\;energy_{oil}}{Yield_{meal}\;*\;energy_{meal\;}+\;Yield_{oil}\;*\;energy_{oil}}\right)$$

3. Freshwater fish-based ingredients
  a. Fresh fishes
    For freshwater fish-based ingredients, two levels of NPPU were calculated for each species:
    — Generic NPPU (Tiers 1):
      ○ Generic TE: TE = 10 % [15].
      ○ Generic carbon content: The carbon content of 1 kg of fish is 111.1 g [16].
      ○ Specific TL [3]
    — Semi-specific NPPU (Tiers 2):
      ○ Generic TE: TE = 10 % [15] (in absence of specific data).
      ○ Specific carbon content: This can differ from a fish to another and is not equal to the generic 111.1 g/kg value. Specific values from literature were used [[32], [33]].
      ○ Specific TL [3]
  b. Fishmeal and fish oil
    NPPU have been calculated for one kilogram of fishmeal and fish oil using mass and energetic allocations, as for marine fish-based ingredients. In absence of specific yields and energy densities, generic values were used.
4. Other animal-based ingredients
  a. Fresh whole bodies
    For other animals, one tiers of NPPU was calculated:
    ○ Generic TE: TE = 10 %
    ○ Specific carbon content from literature [[34], [35], [36], [37], [38], [39], [40]]
    ○ Specific TL from literature and own estimations [41,42].
  b. Ingredients
    NPPU have been calculated for one kilogram of animal-based ingredients using mass allocation. It consisted in choosing among the different fresh items, the appropriate one for the ingredient level (e.g., pig slaughtered at 110 kg for pig by-products).
5. Milk-based ingredients
  For the ingredients based on milk and by-products (e.g., whey), the composition was extracted from Feedtables and a Trophic Level of 2 was applied, according to Bonhommeau et al. [41].
6. Uncertainty analysis

The uncertainty related to NPPU values was quantified at the specie-level, by applying the error propagation techniques based on partial derivatives. We computed partial derivatives with respect to each input variable (C_content, TE and TL) then applied first-order Taylor expansion (formula 5):

(5) $$\sigma_{NPPU}^{2}={\left(\frac{\partial NPPU}{\partial C}*\sigma_{C}\right)}^{2}+{\left(\frac{\partial NPPU}{\partial TE}*\sigma_{TE}\right)}^{2}+{\left(\frac{\partial NPPU}{\partial TL}*\sigma_{TL}\right)}^{2}$$

This allowed to compute standard deviation of the NPPU values available in NPPU_SD.csv file.

## Limitations and recommendations

Limitations of the published dataset are listed below:

1. Methodological limitations:
  - Differences in methods: some NPPU are calculated for fresh animals then values for ingredients are generated applying allocation factors. For plant-based and milk-based ingredients, NPPU are directly calculated using the composition of the final ingredients. For plant-based ingredients, this is due to the fact that those compositions are not available for the whole plant, but at the ingredient level. For milk-based ingredients, this is due to the lack of appropriate data for now, making difficult to allocate the NPPU of one kilogram of milk to all possible coproducts through complex and diverse value chains [43].
  - For some calculations (depending on the specificity level), specific TEs were used. However, they were considered not varying between trophic levels for a given ecosystem.
  - Trophic levels have been extracted from the FishBase database using rfishbase, giving an average TL by specie. They are specific to one specie but independent from the location or size of the catch.
  - In regards with the price volatility, it has been chosen to not provide NPPU of ingredients using economic allocation, although this is the most used, referring to Cashion et al. [6]. However, values are proposed in other publications (in particular [11]). The final user can easily apply its own economic values to the NPPU at the animal-level provided in the present paper, notably in the objective to follow PEFCR recommendations for feed.
2. Other limitations:
  - Diversity of literature data used: when it was possible, efforts have been made to use homogeneous data, coming from a restricted range of database or studies. However, in some cases, it was necessary to multiply the sources, bringing possible variability in data accuracy, robustness and protocols.
  - Non exhaustivity: if this dataset provides a wide range of NPPU values, it has no objective to be exhaustive and could be updated in the future.
  - Some NPPU values showed very high standard deviation values, especially those with high trophic levels. Users should use these values carefully and recalculate their own NPPU values if they have more certain values for the input parameters.

Therefore, some recommendations are provided with the dataset:

- Specific NPPU calculation: when possible, it is recommended to calculate NPPU specifically to the system under study.
- If using NPPU from this dataset, users should prefer NPPU calculated on fresh animals then apply their own allocation methods.
- Same methodology should be preferred in a single study, enabling comparison between scenarios/systems/feed formulations.

## Ethics Statement

The authors confirm that the current work does not involve human subjects, animal experiments, or any data collected from social media platforms.

## CRediT Author Statement

**SLF:** Methodology, Data curation, Writing – original draft preparation, **AW:** Methodology, Data curation, Writing – review & editing, **JA:** Methodology, Data curation, Writing – review & editing.

The authors thank their colleagues from INRAE UMR SAS for fruitful discussions, in particular Hayo Van der Werf.

## Data Availability

https://entrepot.recherche.data.gouv.fr/.
